# The desmosomal cadherin Desmoglein-2 controls extracellular matrix expression and remodeling via NF-κB signaling in keratinocytes

**DOI:** 10.3389/fcell.2025.1691260

**Published:** 2025-10-30

**Authors:** Sadie E. Hunter, Zara T. Patel, Beatriz Mateo, Joshua M. Powers, Austin Y. Shull, Adi D. Dubash

**Affiliations:** ^1^ Department of Biology, Furman University, Greenville, SC, United States; ^2^ Department of Biology, Presbyterian College, Clinton, SC, United States

**Keywords:** desmoglein 2, desmosome, cadherin, extracellular matrix (ECM), keratinocyte, wound healing, cell migration, NF-KappaB

## Abstract

Desmogleins are transmembrane cadherin proteins and obligate members of the desmosome, a cell-cell adhesion complex which connects adjacent cells and provides structural integrity to tissues. While Desmogleins are well-known for their importance in maintenance of cell-cell junctions, several studies have also highlighted their role in signaling crosstalk with cell-matrix adhesions and the extracellular matrix (ECM). We have recently shown that Desmoglein-2 (Dsg2) controls cell spreading on ECM substrates (fibronectin and collagen) and phosphorylation of focal adhesion proteins via Rap1 GTPase signaling. In our current study, we show that loss of Dsg2 in keratinocytes enhances the expression of ECM proteins and matrix metalloproteinases (MMPs), an effect that was not recapitulated upon loss of Desmocollin-2 (Dsc2) or loss of Dsg2 in other epithelial cell types. Signaling pathways well-known to control ECM function (TGF-β and Rho) were not involved in Dsg2-mediated changes in ECM gene expression, but an analysis of global transcriptome changes by RNA sequencing identified major changes in Nuclear Factor-kappa B (NF-κB)-mediated signaling in Dsg2-deficient cells. Interestingly, NF-κB (RelA) activation is elevated in Dsg2-deficient cells, and knockdown of RelA rescued both the enhanced expression of ECM/MMP genes and the enhanced migratory ability of Dsg2-deficient cells. Taken together, this study has identified an important link between Dsg2 and NF-κB signaling involved in controlling matrix production and remodeling, which has relevance for multiple processes in the epidermis such as wound healing and psoriasis.

## Introduction

The cadherins Desmoglein (Dsg) and Desmocollin (Dsc) are single-pass transmembrane proteins present within the desmosome cell-cell adhesion complex (Delva et al., 2009; Thomason et al., 2010). Dsg and Dsc cadherins form heterophilic and homophilic interactions in the extracellular space. On the intracellular side, armadillo proteins Plakophilin (Pkp) and Plakoglobin (Pg) bind to the cytoplasmic tails of the desmosomal cadherins and provide further connections to Desmoplakin (Dsp), which links the cytoplasmic plaque to the intermediate filament network (Delva et al., 2009; Thomason et al., 2010; Bharathan et al., 2024). Linkage of the transmembrane desmosome complex to the intermediate filament network allows the desmosome to perform its main functions of cell-cell adhesion, maintenance of tissue structure, shock absorption and resistance to mechanical strain. Although desmosomal cadherins are typically studied for their role in maintenance of desmosome structure and cell-cell adhesion, different Dsg and Dsc isoforms have been shown to have cellular functions involved in a range of different biological processes such as cell differentiation, proliferation, migration, mechano-transduction, wound healing and apoptosis (El-Amraoui and Petit, 2010; Hegazy et al., 2021; Min et al., 2023). Loss of desmosomal cadherins results in a variety of different skin blistering disorders (such as pemphigus), arrhythmogenic cardiomyopathy (characterized by junctional defects, cardiomyocyte death and myocardial fibrosis) and various forms of cancer (Amagai and Stanley, 2012; Basso et al., 2012; Hegazy et al., 2021; Engel et al., 2024).

Our prior work has shown that loss of Desmoglein-2 (Dsg2), but not Desmocollin-2 (Dsc2), results in increased spreading of cells on extracellular matrix proteins (fibronectin and collagen) and enhanced phosphorylation of focal adhesion proteins FAK and Paxillin (Shelton et al., 2021). The effect of Dsg2 on cell spreading was tied to changes in Rap1 GTPase-mediated signaling, as Rap1 activity was elevated in cells lacking Dsg2, and inhibition of Rap1 in these cells rescued the observed changes in cell spreading and focal adhesion protein phosphorylation. Based on these prior experiments showing Dsg2’s effects on cell-matrix attachment and focal adhesion signaling, we sought to investigate whether Dsg2 also controls the expression of ECM components themselves, the signaling pathways involved, and whether these molecular changes translate into altered cellular behaviors relevant to wound healing.

ECM proteins such as collagen and fibronectin provide tissues with both structural support and signaling cues relevant to developmental processes, normal tissue structure and wound healing (MacCarthy-Morrogh and Martin, 2020; Walma and Yamada, 2020; Potekaev et al., 2021). We show here that loss of Dsg2 results in upregulated expression of key ECM proteins, as well as matrix metalloproteinases (MMPs), enzymes responsible for ECM degradation and remodeling (Li et al., 2000; Bonnans et al., 2014). The coordinated regulation of ECM production and remodeling by MMPs is particularly critical for controlling cell migration during both physiological processes like wound repair and pathological conditions such as cancer. While several signaling pathways are known to control ECM gene expression, the specific mechanisms by which desmosomal cadherins regulate these processes remain poorly understood.

To address this knowledge gap, we employed a combination of targeted gene expression analysis and unbiased transcriptome approaches to identify the signaling pathways responsible for Dsg2-mediated ECM regulation. Here we demonstrate that Dsg2 controls ECM and MMP gene expression through a previously unrecognized connection to NF-κB, revealing a novel mechanism by which cell-cell adhesion proteins coordinate matrix remodeling and cell migration in keratinocytes. NF-κB is a transcription factor which controls a wide range of gene expression programs related to inflammation, proliferation and epithelial to mesenchymal transition (Hayden and Ghosh, 2008; Oh et al., 2023). In the canonical NF-κB signaling pathway, dimers of the Rel family of proteins (prototypically RelA/p65 and p50) are kept in an inactive state in the cytoplasm via association with IκB proteins (Moynagh, 2005). A wide range of upstream signals can result in activation of upstream IκB kinases (IKKα/β) which phosphorylate and induce degradation of the inhibitory IκB protein, thereby allowing for nuclear accumulation of NF-κB and transcriptional activation of downstream genes (Moynagh, 2005; Hayden and Ghosh, 2008; Mitchell et al., 2015).

Our results show a marked elevation in NF-κB signaling upon loss of Dsg2, as well as a rescue of Dsg2 effects on ECM gene expression and keratinocyte migration upon inhibition of NF-κB signaling. Taken together, these findings have important implications for understanding how desmosomal cadherins control epidermal homeostasis and may provide new therapeutic targets for inflammatory skin diseases and wound healing disorders.

## Materials and methods

### Cell culture, siRNA transfections and drug treatments

A431, HaCaT and MCF7 cells were cultured in DMEM with 10% Fetal Bovine Serum and antibiotic/antimycotic solution (Corning). SCC9 cells were cultured in DMEM and Ham’s F-12 50/50 mix with 10% Fetal Bovine Serum and antibiotic/antimycotic solution. Control and Dsg2 knockout A431 cells were a kind gift from Dr. Daniel Conway (Ohio State University) and were generated via CRISPR/Cas9 editing (Baddam et al., 2018). For siRNA-mediated knockdown, cells at ∼20–40% confluency were transfected with either control or gene-specific siRNA using DharmaFECT1 (Dharmacon) according to manufacturer’s protocols, followed by harvesting cells for subsequent analysis at 3–4 days post siRNA transfection. DsiRNA sequences (purchased from Integrated DNA Technologies) used in this study are as follows: Dsg2 (CUCAGCAUCUUCAAGAUGUACCUTA), Dsc2 (GUGAGAGAUAGACUUGGCAUGUCTA), NF-κB (AUAUGAGACCUUCAAGAGCAUCATG). Where indicated, cells were treated with the following drugs purchased from MilliporeSigma: BMS345541 (1 µM), Batimastat (1 µM), SB431542 (5 µM), GGTI-298 (10 µM), Y27632 (1 µM), CCG1423 (1 µM).

### Wound healing assays

For wound healing assays, cells were grown to confluence within culture inserts (Ibidi). Lifting of the culture insert results in the creation of a uniform wound area in all samples (see Supplementary Figure S2, 0 hr). After two washes with Phosphate Buffered Saline (PBS) to remove cell debris, the cells were returned to media containing 10 μg/mL Mitomycin C (Sigma) to eliminate proliferation-dependent effects on wound closure. At 12–16 hr post-wounding, cells were fixed and stained with AlexaFluor 568-tagged Phalloidin. An EVOS M7000 inverted fluorescence microscope was used to generate images of the entire remaining wound area for each sample. Remaining wound area was quantified via area measurements on thresholded images in FIJI. Graphs shown represent fold change measurements of remaining wound area from representative experiments, and statistical analysis was performed as described below.

### Immunofluorescence and microscopy

Cells on coverslips were fixed in 4% paraformaldehyde (Electron Microscopy Sciences) for 15–30 min, washed 3 times with PBS, permeabilized with 0.3% Triton X-100 for 10 min (MilliporeSigma), washed 3 times with PBS, and blocked with 5% normal donkey serum (Jackson Immunoresearch) for 60 min at 37 °C. Coverslips were incubated with DAPI, AlexaFluor 568-tagged Phalloidin (ThermoFisher) or primary and secondary antibodies (listed in Supplementary Table S1) and mounted in Prolong Gold (ThermoFisher). To visualize decellularized matrix, control and Dsg2 knockdown SCC9 samples growing on coverslips were decellularized as described in (Harris et al., 2018). Briefly, coverslips were washed 2 times with PBS and then 3 times in a buffer containing 100 mM Na_2_HPO_4_, 2 mM MgCl_2_ and 2 mM EGTA (pH 9.6). Cells were lysed by incubation in a buffer containing 8 mM Na_2_HPO_4_ and 1% Triton X-100 (pH 9.6) for 30 min at 37 °C along with gentle shaking. Finally, samples were washed 4 times in a buffer containing 10 mM Na_2_HPO_4_ and 100 mM KCl (pH 7.5). Decellularized matrix remaining on the coverslip was fixed in 4% paraformaldehyde, and samples processed for immunofluorescence as described above. Immunofluorescence images shown in Figures 2, 5 were taken using a Leica Microsystems TCS SP8 Spectral Confocal Microscope, an oil immersion ×63 objective (HC PL APO ×63/1.40 OIL CS2) and Leica LAS X SP8 control software. All images shown are representative of three or more experiments, with 10–20 random fields of view imaged per sample per experiment using the same exposure time and camera settings.

### Western blots

To analyze protein expression levels, cell samples were washed briefly in PBS, lysed in urea sample buffer (8 M deionized urea, 1% SDS, 10% Glycerol, 60 mM Tris pH 6.8, and 5% β-mercaptoethanol) and equalized for total protein concentration using a Take3 plate and a Synergy LX plate reader (Biotek). Samples were subjected to SDS-PAGE along with a pre-stained protein ladder (PureView, Azura Genomics), followed by transfer to PVDF membranes (Immobilon-P_SQ_, MilliporeSigma). Membranes were probed with primary and secondary antibodies (listed in Supplementary Table S1) and washed in Tris Buffered Saline +0.2% Tween. Blots were visualized by enhanced chemiluminescence using Immobilon Crescendo Western HRP Substrate (MilliporeSigma) and a ChemiDoc Imaging System (BioRad). For densitometry analysis, Fibronectin protein band intensity was measured in FIJI (integrated density measurement) and normalized to tubulin protein band intensity. Average fold change in normalized Fibronectin protein intensity was calculated across three independent experiments. All western blots shown are representative of data from three or more independent experiments.

### ELISA assays

MMP1 levels in cell culture supernatant were assayed using the Human MMP-1 ELISA Kit (Thermo Fisher Scientific) according to manufacturer’s instructions. Briefly, samples of cell culture supernatant were taken from control A431 cells (A431CT) and Desmoglein-2 knockout (Dsg2KO) A431 cells, followed by a brief centrifugation (2000 x g for 10 min) to pellet any cellular debris. Following the ELISA procedure on A431CT and Dsg2KO supernatant samples, absorbance was measured at 450 nm using a Synergy LX plate reader (Biotek). Blank absorbances were subtracted from all data points, followed by normalization to total protein levels (measured by lysis of cells in the same wells from which supernatant samples were collected). ELISA assays shown are representative of data from three independent experiments.

### Quantitative real-time PCR

To measure mRNA transcript levels via quantitative real-time PCR (qPCR), RNA was isolated using the RNA Mini kit, according to manufacturer’s instructions (Zymo). Total RNA concentrations were equalized between samples using a Take3 plate and a Synergy LX plate reader (Biotek), and qPCR was performed using gene-specific primers and RNA-to-C_T_ 1-step kit (Azura Genomics). qPCR cycling was performed in a QuantStudio3 instrument (Applied Biosystems). All gene-specific primers used for qPCR in this study are listed in Supplementary Table S2. Calculations for relative mRNA levels were performed using the ΔΔC_T_ method, normalized to GAPDH, and represented as fold change values compared with control samples. All graphs for mRNA levels shown were obtained from quantification of three or more independent biological replicates, and statistical analysis was performed as described below.

### RNA sequencing analysis

Total RNA from control A431 (A431CT) and A431 Dsg2 knockout (Dsg2KO) cells were isolated using TRIzol. Next, concentration and quality of isolated RNA was first assessed using NanoDrop (260/280 and 260/230 ratios >1.8) with RNA quality being further validated using an Agilent Bioanalyzer 2,100. Libraries were prepared sequenced on an Illumina platform using paired-end sequencing (2 × 150 bp) (Novogene). Raw sequencing reads were processed using CLC Genomics Workbench (version 23, Qiagen). Quality control was performed to remove low-quality reads and adapter sequences, and cleaned reads were aligned to GRCh38/hg38 using the RNA-Seq Analysis pipeline within CLC Genomics Workbench. Gene expression levels were quantified as transcripts per million (TPM), and differential gene expression analysis was performed using the built-in statistical tools with genes being considered differentially expressed with an adjusted Benjamini-Hochberg FDR p-value <0.05 and a log2 fold change of ±0.5. Overrepresentation gene ontology analysis was performed on differentially expressed genes using the Gene Hallmarks gene sets from the Molecular Signatures Database (MSigDB) through the Gene Set Enrichment Analysis (GSEA) platform to identify enriched biological pathways and processes. The RNA sequencing data reported in this study have been deposited in NCBI’s Gene Expression Omnibus (GEO) database under accession number GSE303980.

### Statistical analysis

For all experiments shown, data was obtained from a minimum of three independent experiments/biological replicates. In many cases, multiple technical replicates (wells, coverslips, etc.) were processed in parallel within each independent experiment. Representative images/blots are displayed in all figures, and graphs for each experiment are indicated in the figure legends as either mean ± standard deviation (s.d.) or mean ± standard error of the mean (s.e.m.). All statistical analysis was performed using Prism 10 (GraphPad). For data comparing two conditions, statistical analysis was performed using a student’s two-tailed t-test. For data comparing three or more conditions, statistical analysis was performed using Welch’s ANOVA followed by Dunnett T3 analysis. Statistical significance was represented in graphs as follows: N.S. = not significant, * = p < 0.05, ** = p < 0.01, *** = p < 0.001.

## Results

### Loss of Dsg2, but not Dsc2, enhances extracellular matrix gene expression

**FIGURE 1:**
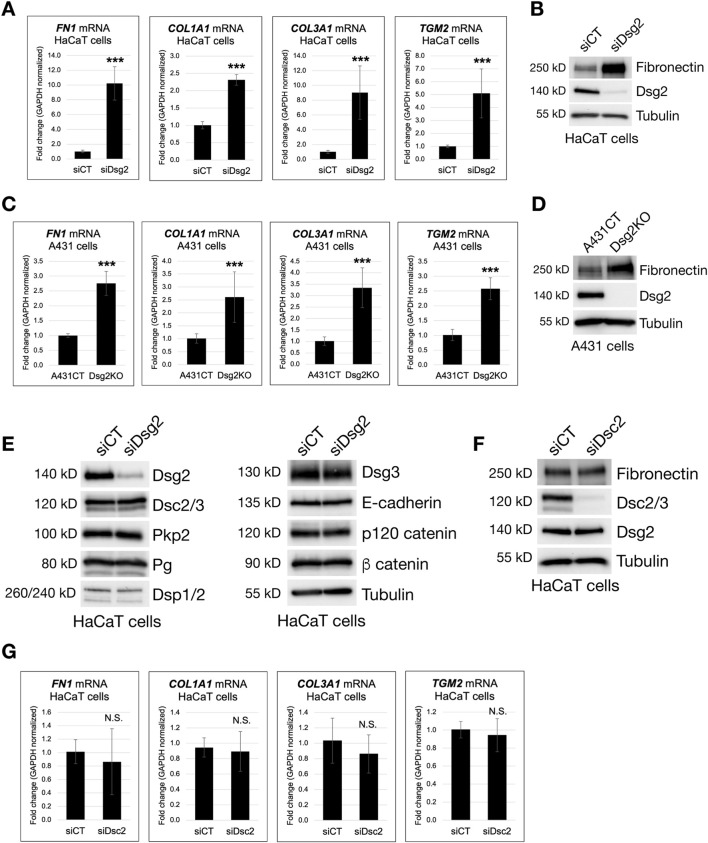
Loss of Dsg2, but not Dsc2, enhances extracellular matrix gene expression. **(A)** HaCaT cells were transfected with control siRNA (siCT) or siRNA specific for Desmoglein-2 (siDsg2). 72 h post transfection, total RNA was isolated from cell samples, followed by qPCR to analyze mRNA levels of the following genes: Fibronectin (*FN1*), Collagen Type I α1 (*COL1A1*), Collagen Type III α1 (*COL3A1*) and Transglutaminase 2 (*TGM2)*. **(B)** HaCaT cells expressing either siCT or siDsg2 were processed for SDS-PAGE and blotted for the following proteins: Fibronectin, Dsg2 and Tubulin (loading control). **(C)** Total RNA was isolated from control A431 cells (A431CT) and Desmoglein-2 knockout (Dsg2KO) cells, followed by qPCR to analyze mRNA levels of *FN1*, *COL1A1, COL3A1* and *TGM2*. **(D)** A431CT and Dsg2KO cells were processed for SDS-PAGE and blotted for the following proteins: Fibronectin, Dsg2 and Tubulin (loading control). **(E)** siCT and siDsg2 HaCaT cells were processed for SDS-PAGE and blotted for the following proteins: Dsg2, Desmocollin-2/3 (Dsc2/3), Plakophilin-2 (Pkp2), Plakoglobin (Pg), Desmoplakin-1/2 (Dsp1/2), Desmoglein-3 (Dsg3), E-cadherin, p120 catenin, β catenin and Tubulin (loading control). **(F)** HaCaT cells were transfected with control siRNA (siCT) or siRNA specific for Desmocollin-2 (siDsc2). 72 h post transfection, samples were processed for SDS-PAGE and blotted for the following proteins: Fibronectin, Dsc2/3, Dsg2 and Tubulin (loading control). **(G)** Total RNA was isolated from siCT or siDsc2 HaCaT cells, followed by qPCR to analyze mRNA levels of *FN1*, *COL1A1*, *COL3A1* and *TGM2*. All graphs shown represent fold change values of mRNA levels, with error bars indicating s.d. (N.S. = not significant, *** = p < 0.001).

To analyze a role for desmosomal cadherins in regulation of ECM gene expression, we first employed an siRNA-mediated knockdown approach in HaCaT keratinocytes, followed by analysis of transcriptional changes by quantitative PCR. Based on prior observed changes in cell-matrix attachment, cell spreading and focal adhesion formation in Dsg2-deficient cells, we hypothesized that Dsg2 knockdown would result in enhanced expression of ECM proteins. Compared to HaCaT cells expressing control siRNA (siCT), Dsg2 knockdown cells (siDsg2) demonstrated elevated mRNA levels of several different ECM genes, Fibronectin (*FN1*), Collagen Type I α1 (*COL1A1*) and Collagen Type III α1 (*COL3A1*) (Figure 1A). We also observed an increase in expression of the Transglutaminase 2 gene (*TGM2)* upon Dsg2 knockdown, which is a β-integrin-binding cell-surface co-receptor for Fibronectin which plays many different roles in adhesion, migration and ECM organization (Akimov et al., 2000; Nurminskaya and Belkin, 2012).

Analysis of Desmoglein-2 knockout (Dsg2KO) A431 cells showed similar increases in *FN1*, *COL1A1, COL3A1* and *TGM2* mRNA compared to control A431 cells (A431CT), showing that these changes occur in several different keratinocyte cell lines (Figure 1C). Western blots in both HaCaT and A431 cells confirmed that Dsg2-deficient keratinocytes also have elevated Fibronectin (FN) protein levels compared to control cells (Figures 1B,D). To confirm that these changes were not caused by loss of other cell-cell junction proteins in Dsg2-deficient cells, we performed western blots for a range of different adherens junction and desmosomal proteins. Dsg2 knockdown in HaCaT cells did not significantly perturb the expression of other cadherins (E-cadherin, Dsg3 or Dsc2) or other junctional components (p120 catenin, β catenin, Pkp2, Pg and Dsp1+2) (Figure 1E). Moreover, knockdown of Dsc2 in HaCaT cells did not elicit the same increase in ECM expression (either at the mRNA or protein level), indicating that this effect is specific to loss of Dsg2 (Figures 1F,G).

### Dsg2-deficient cells have enhanced matrix deposition and matrix metalloproteinase expression

**FIGURE 2:**
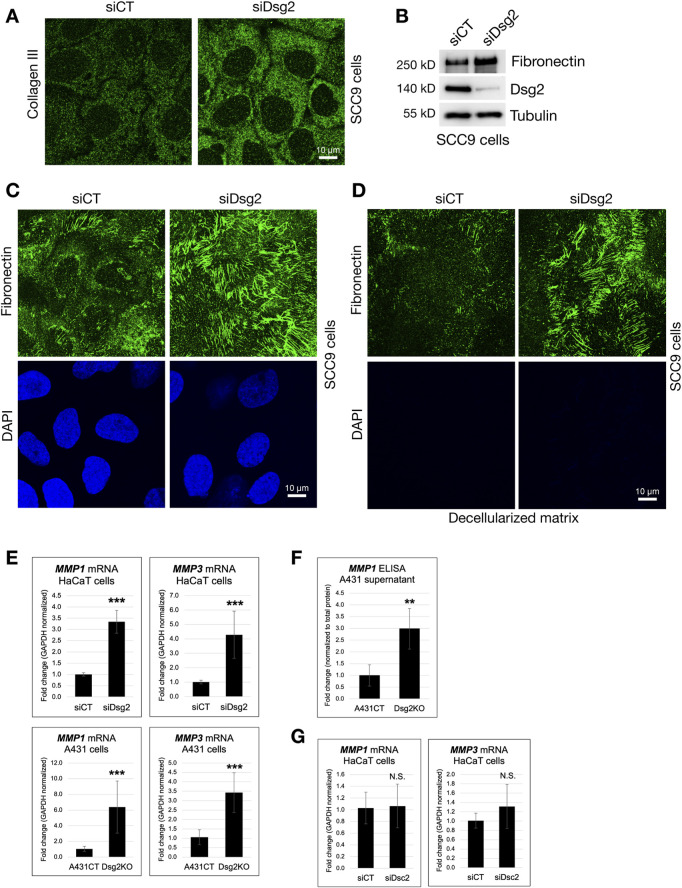
Dsg2-deficient cells have enhanced matrix deposition and matrix metalloproteinase expression. **(A,C)** SCC9 cells growing on coverslips were transfected with control siRNA (siCT) or siRNA specific for Desmoglein-2 (siDsg2). 72 h post transfection, cells were fixed, processed for immunofluorescence and stained with an anti-Collagen III **(A)** or anti-Fibronectin antibody **(C)**. Coverslips were imaged with a Leica Microsystems TCS SP8 Spectral Confocal Microscope (scale bar = 10 μm). **(B)** siCT and siDsg2 expressing SCC9 cells were processed for SDS-PAGE and blotted for Fibronectin, Dsg2 and Tubulin (loading control). **(D)** siCT and siDsg2 expressing SCC9 samples were decellularized (as described in methods), and deposited matrix was fixed and stained with an anti-Fibronectin antibody. Decellularization was confirmed via loss of DAPI staining. **(E)** HaCaT cells were transfected with control siRNA (siCT) or siRNA specific for Desmoglein-2 (siDsg2). 72 h post transfection, total RNA was isolated from cell samples, followed by qPCR to analyze mRNA levels of Matrix Metallopeptidase 1 (*MMP1*) and Matrix Metallopeptidase 1 (*MMP3*). Identical experiments for MMP1 and MMP3 mRNA levels were performed in A431CT and Dsg2KO cells. All graphs shown represent fold change values of mRNA levels, with error bars indicating s.d. ( *** = p < 0.001). **(F)** MMP1 protein levels in cell culture supernatant from A431CT and Dsg2KO cells were analyzed via an ELISA assay. Graph shows fold change in MMP1 levels in A431 cell culture supernatant normalized to total cellular protein. **(G)** Total RNA was isolated from HaCaT cells transfected with siCT or siDsc2, followed by qPCR to analyze mRNA levels of *MMP1 and MMP3*. Graphs shown represent fold change values of mRNA levels, with error bars indicating s.d. (N.S. = not significant).

To visualize extracellular matrix, we fixed SCC9 keratinocytes expressing either control (siCT) or Dsg2-specific (siDsg2) siRNA, followed by staining with either an anti-Collagen III or anti-Fibronectin antibody. Compared to control cells, increased staining for both Collagen III and Fibronectin was observed in Dsg2-deficient SCC9 cells via immunofluorescence (Figures 2A,C). As seen in HaCaT and A431 keratinocytes, elevation of Fibronectin protein levels in SCC9 cells upon loss of Dsg2 was observed via Western blot (Figure 2B). To determine if the increased ECM expression seen in Dsg2-deficient cells results in enhanced secretion and deposition of ECM, we decellularized control and Dsg2-deficient SCC9 samples on coverslips (confirmed via loss of DAPI staining). Fixation and staining of decellularized matrix with an anti-Fibronectin antibody confirmed that ECM deposition is also elevated upon Dsg2 knockdown (Figure 2D).

Matrix metalloproteinases (MMPs) are enzymes important for regulating the composition and structure of the ECM, and they play varied roles in a range of different biological processes such as cell adhesion, growth, migration and the morphogenesis of different tissues. We show that loss of Dsg2 in either HaCaT or A431 cells enhances the expression of the genes Matrix Metallopeptidase 1 (*MMP1*) and Matrix Metallopeptidase 3 (*MMP3*) (Figure 2E). Analysis of MMP1 levels in samples of cell culture supernatant also demonstrated an increase in MMP1 protein expression upon loss of Dsg2 in A431 cells (Figure 2F). As seen for ECM gene expression, Dsc2 knockdown in HaCaTs did not produce increases in MMP gene expression, showing that this effect is specific for loss of Dsg2 (Figure 2G). Lastly, Dsg2 knockdown in another epithelial cell type (MCF7) did not cause an increase in either ECM or MMP gene expression, suggesting that loss of Dsg2 may not cause these changes in other epithelial cell types (Supplementary Figures S1A,B). Taken together, these data highlight the important role for Dsg2 in controlling ECM expression and remodeling in keratinocytes.

### The enhanced migration of Dsg2-deficient keratinocytes is dependent on MMP activity

**FIGURE 3:**
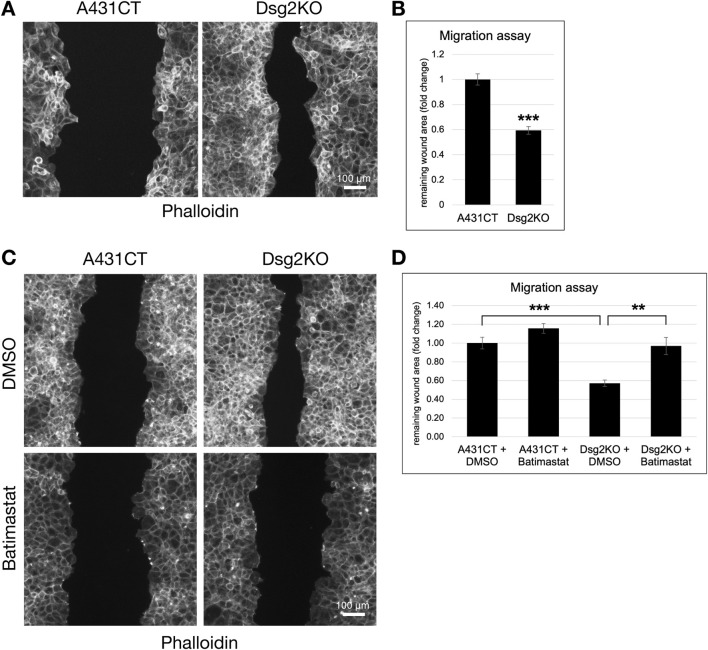
The enhanced migration of Dsg2-deficient keratinocytes is dependent on MMP activity. **(A)** Control A431 cells (A431CT) and Dsg2 knockout cells (Dsg2KO) were grown to confluency within culture inserts (Ibidi). 12–16 h after lifting the insert, cells were fixed and stained with AlexaFlour568-Phalloidin. Remaining wound area was captured with an EVOS M7000 inverted microscope and wound area quantified in FIJI (scale bar = 100 μm). **(C)** A431CT or Dsg2KO cells were grown to confluency within 2- or 3-well culture inserts (Ibidi). Following lifting of the culture insert, cells were treated with either DMSO (vehicle control) or Batimastat (1 µM). 12–16 h later, cells were fixed and with AlexaFlour568-Phalloidin. Remaining wound area was captured with an EVOS M7000 inverted microscope and wound area quantified in FIJI (scale bar = 100 μm). **(B,D)** Graphs shown represent fold change values of average remaining wound area (fold change), with error bars representing s.e.m. ( ** = p < 0.01, *** = p < 0.001). Note that a decrease in remaining wound area is indicative of an increase in rate of cell migration.

The modulation of ECM and MMP gene expression by Dsg2 suggests an important role for Dsg2 in controlling keratinocyte migration. To determine this, we performed wound healing migration assays in A431CT and Dsg2KO cells. Cells were grown to confluence in cell culture inserts, followed by lifting of the culture inserts to create a uniform wound area in all samples (Supplementary Figure S2). A statistically significant decrease in remaining wound area at 12 h post-wound formation showed that Dsg2KO cells close wounds faster than control A431 cells (Figures 3A,B). We hypothesized that changes in MMP gene expression have a causative role in the enhanced migration of Dsg2-deficient keratinocytes. To test this, we performed additional migration assays in A431CT and Dsg2KO cells in the presence of the broad-spectrum MMP inhibitor Batimastat. These assays showed that Batimastat treatment was able to rescue the enhanced migration (or wound closure) seen in Dsg2KO cells (Figures 3C,D). Taken together, these data indicate a causative role for Dsg2-mediated control of MMPs in regulation of cell migration.

### TGF-β, Rap1 or RhoA/SRF signaling does not control ECM gene expression via Dsg2

In order to investigate the mechanisms via which Dsg2 controls ECM and MMP gene expression, we analyzed a potential role for either Transforming Growth Factor-β (TGF-β) or Rho/Serum Response Factor (SRF) signaling, as they are well-known modulators of ECM gene expression and have been shown in prior studies to be altered by loss of other desmosomal proteins such as Pkp2 and Dsp (Godsel et al., 2010; Dubash et al., 2016; Meng et al., 2016; Distler et al., 2019). We have also previously shown that Rap1 and TGF-β2 signaling is required for alterations in focal adhesion formation and cell spreading seen in Dsg2-deficient cells (Shelton et al., 2021). However, inhibition of TGF-β signaling (with SB431542), Rap1 signaling (with GGT1-298), Rho signaling (with Y27632), or SRF signaling (with CCG1423) were all unable to rescue the elevation in fibronectin gene expression seen in Dsg2-deficient cells (Supplementary Figure S3). These data prompted us to undertake an unbiased global approach to analysis of Dsg2-mediated changes in keratinocytes.

### Loss of Dsg2 in keratinocytes causes an upregulation of inflammatory expression signatures

**FIGURE 4:**
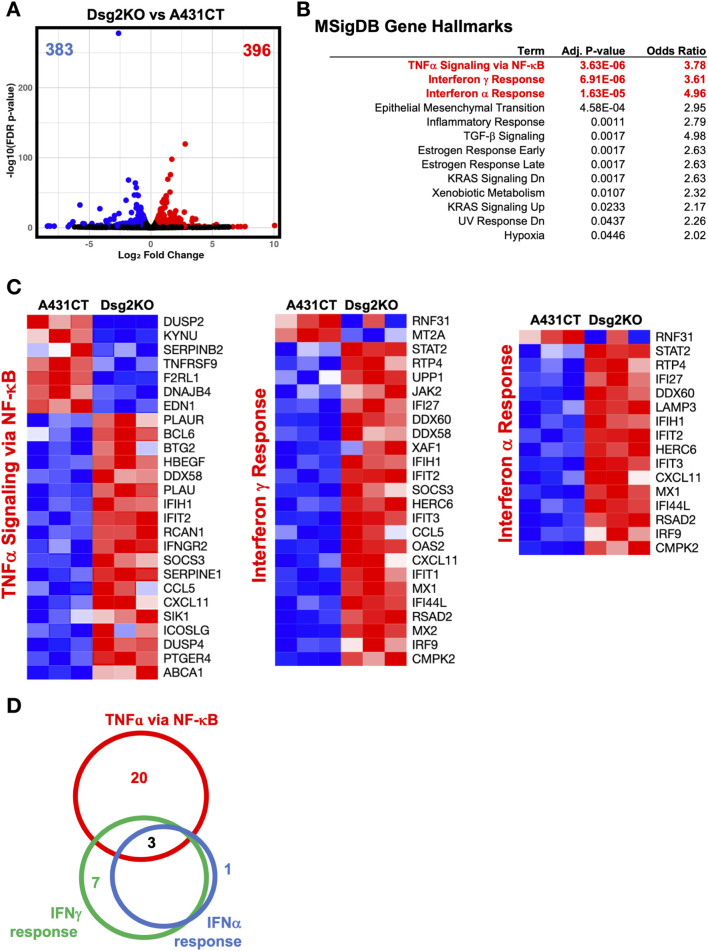
Loss of Dsg2 in keratinocytes causes an upregulation of inflammatory expression signatures. **(A)** Volcano plot showing differential gene expression between Dsg2KO and A431CT cells. Blue dots represent downregulated genes (n = 383), red dots represent upregulated genes (n = 396), gray dots represent non-significant genes. **(B)** Gene set enrichment analysis using MSigDB hallmark gene sets showing enriched pathways in Dsg2KO cells. **(C)** Heatmaps displaying expression patterns of genes from TNF-α signaling via NF-κB (left), Interferon γ Response (middle), and Interferon α Response (right) pathways. Red indicates upregulation, blue indicates downregulation. **(D)** Venn diagram illustrating gene overlap between the three major immune pathways, with three genes (IFIT2, IFIH1, CXCL11) common to all pathways. RNA sequencing performed with n = 3 biological replicates per condition; significance set at adjusted p-value <0.05.

In order to identify key cellular pathways that may be involved in the regulation of ECM and MMP gene expression by Dsg2, we decided to broadly investigate transcriptome changes in Dsg2KO cells via RNA sequencing analysis. Differential expression analysis identified 779 significantly altered genes, with 396 genes upregulated and 383 genes downregulated in Dsg2KO cells compared to controls (Figure 4A). Overrepresentation-based gene ontology (GO) analysis using MSigDB hallmark gene sets demonstrated that Dsg2 loss predominantly activated NF-κB-mediated signaling transcripts (Figure 4B) with the most significantly enriched gene sets being Tumor Necrosis Factor-alpha (TNFα) signaling via NF-κB (adj p-value = 3.63E-06, odds ratio = 3.78), followed by interferon γ response (adjusted p-value = 6.91E-06, odds ratio = 3.61), and interferon α response (adjusted p-value = 1.63E-05, odds ratio = 4.96). Additional enriched pathways encompassed epithelial-mesenchymal transition, inflammatory response, and TGF-β signaling.

Pathway-specific heatmaps representing the top three MSigDB Gene Hallmarks confirmed the coordinated upregulation of NF-κB-related inflammatory genes in Dsg2KO cells (Figure 4C). The expression profiles showed consistent activation of genes across multiple immune pathways, with prominent upregulation of classical inflammatory genes like *CCL5*, *CXCL11*, *IFIT2*, *SOCS3*, and *IFIH1*. Notably, *IFIT2*, *IFIH1*, and *CXCL11* were the three genes commonly upregulated across the three MSigDB Gene Hallmark gene sets (Figure 4D). These results demonstrate coordinated upregulation of classical NF-κB target genes, along with activation of other known inflammatory response transcripts, suggesting that Dsg2 may function to suppress NF-κB signaling and its loss can lead to enhanced inflammatory activation.

### Dsg2 knockdown results in activation of NF-κB signaling

**FIGURE 5:**
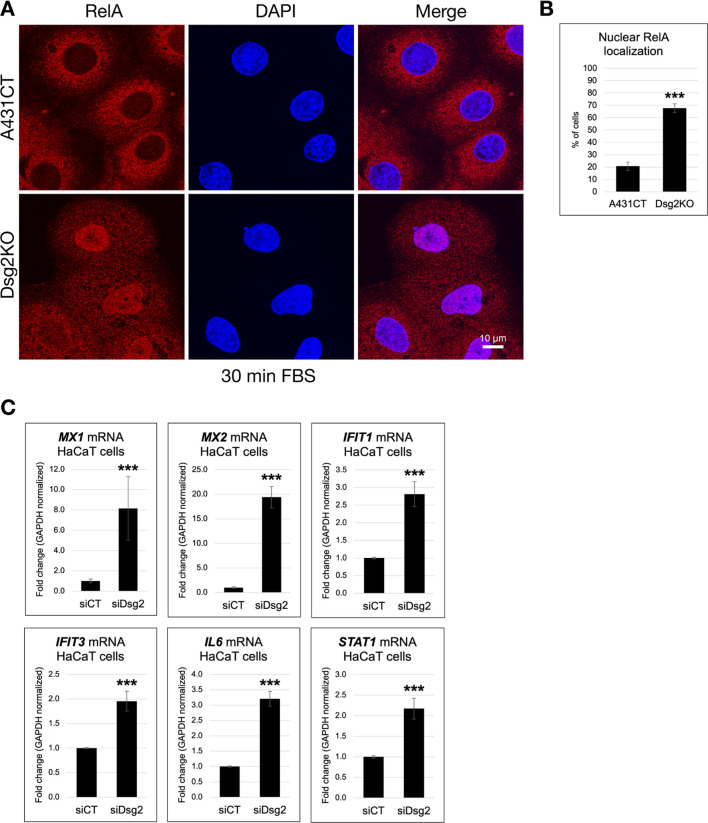
Dsg2 knockdown results in activation of NF-κB signaling. **(A)** A431CT and Dsg2KO cells growing on coverslips were serum-starved overnight, treated with Fetal Bovine Serum (FBS) for 30 min, fixed and processed for immunofluorescence with an anti-RelA antibody and DAPI (to visualize nuclei). Coverslips were imaged with a Leica Microsystems TCS SP8 Spectral Confocal Microscope, and the number of cells with nuclear staining for RelA was scored (scale bar = 10 μm). **(B)** The graph represents the percentage of A431CT and Dsg2KO cells with nuclear localized RelA ( *** = p < 0.001). **(C)** Total RNA was isolated from HaCaT cells transfected with siCT or siDsg2, followed by qPCR to analyze mRNA levels of *MX1, MX2, IFIT1, IFIT3, IL6,* and *STAT1*. Graphs shown represent fold change values of mRNA levels, with error bars indicating s.d. ( *** = p < 0.001).

Based on the results of these RNAseq experiments, we investigated whether NF-κB signaling is altered in Dsg2-deficient keratinocytes. To determine this, we analyzed the nuclear localization of the p65/RelA subunit via immunofluorescence staining. Dsg2KO A431 cells demonstrated a significant increase in nuclear localization of RelA in response to serum stimulation compared to control cells (Figures 5A,B). Moreover, we show that loss of Dsg2 in HaCaT cells triggers a significant increase in a wide range of NF-κB target genes, including *MX1, MX2, IFIT1, IFIT3, IL6*, and *STAT1*, providing supportive data for the results of our RNAseq experiments (Figure 5C). These results were also validated via analysis of mRNA levels of NF-κB target genes in A431CT vs. Dsg2KO cells (Supplementary Figure S4). These changes in NF-κB mediated gene expression are not observed upon Dsg2 knockdown in MCF7 cells, which mirrors the specificity we observed for ECM and MMP gene expression changes seen earlier (Supplementary Figure S1C). Taken together, these data show that loss of Dsg2 in keratinocytes induces activation of the NF-κB signaling pathway.

### Inhibition of NF-κB signaling rescues Dsg2-mediated changes in ECM/MMP gene expression and cell migration

**FIGURE 6:**
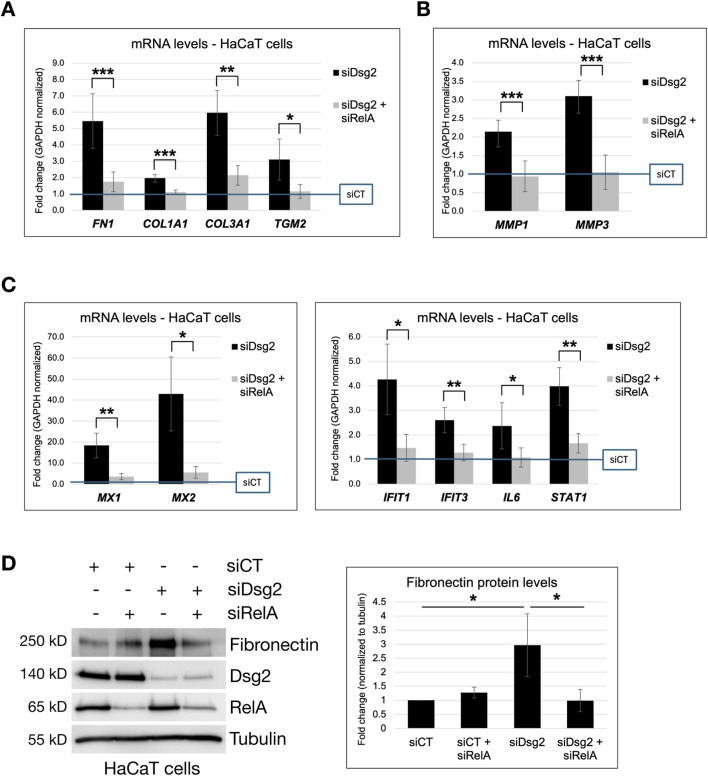
Inhibition of NF-κB signaling rescues Dsg2-mediated changes in ECM and MMP gene expression. **(A–C)** HaCaT cells were transfected with control siRNA (siCT), siRNA specific for Desmoglein-2 (siDsg2) or siRNA specific for RelA/NF-κB (siRelA) as shown. 72 h post transfection, total RNA was isolated from cell samples, followed by qPCR to analyze mRNA levels of *FN1, COL1A1, COL3A1, TGM2, MMP1, MMP3, MX1, MX2, IFIT1, IFIT3, IL6 and STAT1*. Graphs shown represent fold change values of mRNA levels compared to control siCT HaCaT cells (blue reference line), with error bars indicating s.d. ( * = p < 0.05, ** = p < 0.01, *** = p < 0.001). **(D)** HaCaT cells were transfected with control siRNA (siCT), siRNA specific for Desmoglein-2 (siDsg2) or siRNA specific for RelA/NF-κB (siRelA) as shown. 72 h post transfection, samples were processed for SDS-PAGE and blotted for the following proteins: Fibronectin, Dsg2, RelA/NF-kB and Tubulin. The graph shown represents averaged fold change values of Fibronectin protein levels (normalized to tubulin) measured across three independent experiments via densitometry analysis, with error bars indicating s.d ( * = p < 0.05).

To determine if Dsg2 controls ECM gene expression via its effects on NF-κB activity, we blocked NF-κB signaling via knockdown of p65/RelA. Knockdown of both RelA and Dsg2 in HaCaT cells rescued the elevation of ECM genes (*FN1*, *COL1A1, COL3A1* and *TGM2*) seen in Dsg2 knockdown cells alone (Figure 6A). Dsg2-mediated changes in MMP gene expression were also rescued by inhibition of NF-κB signaling using RelA siRNA (Figure 6B). As expected, knockdown of RelA also rescued the elevation of NF-κB target genes (*MX1, MX1*, *IFIT1, IFIT3, IL6* and *STAT1*) seen with Dsg2 knockdown alone, confirming the efficacy of blocking the NF-κB signaling pathway via this approach (Figure 6C). Lastly, knockdown of both RelA and Dsg2 rescued the elevation of FN protein levels as well, compared to Dsg2 knockdown alone (Figure 6D). Taken together, these data confirm a causative role for NF-κB signaling in Dsg2-mediated changes in ECM and MMP gene expression.

**FIGURE 7:**
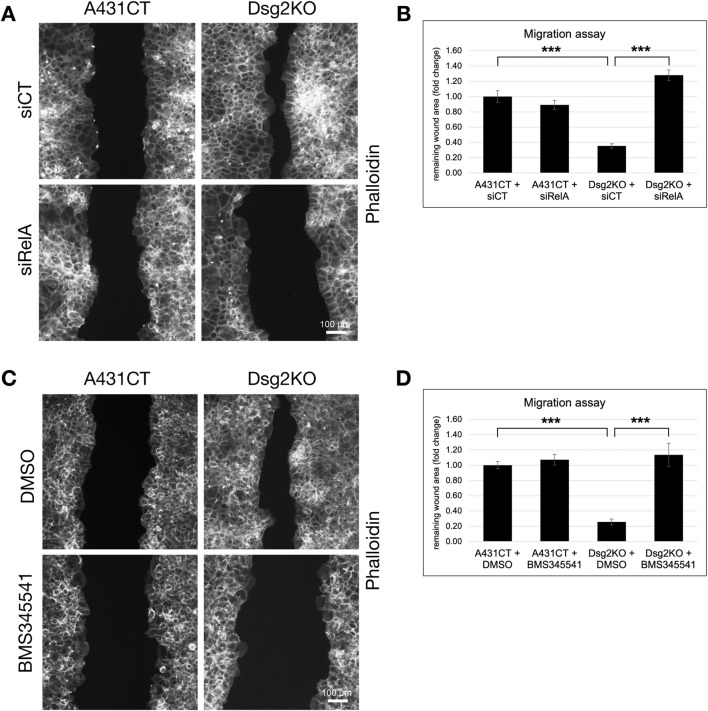
Inhibition of NF-κB signaling rescues Dsg2-mediated changes in cell migration. **(A,B)** A431CT and Dsg2KO cells were transfected with either siCT or siRelA and grown to confluency within culture inserts (Ibidi). 12–16 h after lifting the insert, cells were fixed and stained with AlexaFlour568-Phalloidin (scale bar = 100 μm). **(C,D)** A431CT and Dsg2KO cells were grown to confluency within culture inserts (Ibidi). Following lifting of the culture insert, cells were treated with either DMSO (vehicle control) or BMS345541 (1 µM). 12–16 h later, cells were fixed and with AlexaFlour568-Phalloidin. For all migration assays shown, remaining wound area was captured with an EVOS M7000 inverted microscope and wound area quantified in FIJI (scale bar = 100 μm). Graphs shown represent fold change values of average remaining wound area (fold change), with error bars representing s.e.m. ( *** = p < 0.001). Note that a decrease in remaining wound area is indicative of an increase in rate of cell migration.

We therefore next sought to determine if the ability of Dsg2 to regulate cell migration is due to its effects on NF-κB signaling. Both knockdown of RelA and inhibition of NF-κB effects via the pharmacological inhibitor BMS345541 were able to rescue the enhanced migratory ability of Dsg2-deficient keratinocytes (Figures 7A–D). Taken together, these studies have identified an important link between Dsg2 and NF-κB signaling in the regulation of extracellular matrix composition, remodeling and cell migration.

## Discussion

In connection with their primary adhesive role, desmosomal cadherins are well known for their regulation of epidermal morphogenesis and differentiation (Broussard et al., 2015; Green et al., 2022). Dsg2 null embryos die at implantation, likely due to defects in proliferation of Dsg2-deficient embryonic stem cells (Eshkind et al., 2002). Changes in expression of desmosomal cadherins (from Dsg2 in undifferentiated basal keratinocytes to Dsg1 in suprabasal differentiated keratinocytes) is critically important for coordinating the process of keratinocyte differentiation and maintaining the structure and function of the epidermis (Kowalczyk and Green, 2013).

Considering this critical role for desmosomes in mediating tissue integrity, it is not surprising that mutations in desmosomal cadherins (and other desmosome components) have been linked to a variety of different epidermal disorders (related to skin fragility/blistering, loss of hair, etc.) and also arrhythmogenic cardiomyopathy, which is characterized by loss of cell-cell adhesion and gap junction defects in cardiomyocytes (Delmar and McKenna, 2010; El-Amraoui and Petit, 2010; Amagai and Stanley, 2012; Nitoiu et al., 2014). Besides their central role in maintenance of cell-cell adhesion, desmosomal cadherins have frequently been studied for their ability to coordinate other cellular behaviors (such as proliferation, differentiation, apoptosis, cell shape and migration) via their ability to act as signal transduction hubs (Müller et al., 2021).

Our study identifies a previously unrecognized connection between the desmosomal cadherin Dsg2 and NF-κB signaling that controls ECM gene expression and remodeling in keratinocytes. This discovery reveals a novel mechanism by which cell-cell adhesion proteins coordinate ECM homeostasis through inflammatory signaling pathways, with potential implications for wound healing and skin disease. By demonstrating how Dsg2 regulates ECM and MMP expression via NF-κB activation, our work continues to expand the characterized roles of desmosomal cadherins beyond traditionally described cell-cell adhesion functions.

Following early inflammatory processes in the initial stages of wounding, alterations in MMP activation and FN expression have been described as important aspects of the wound healing process (Potekaev et al., 2021). Prior work showed that a highly invasive A431-III line had significantly high FN and TGM2 levels (with TGM2 acting as a co-receptor with integrins for binding to FN), and that knockdown of either FN or TGM2 reduced migration and invasion of these cells (Chen et al., 2010). There is evidence for the ability of desmosomal proteins to control ECM gene expression, but the effects of specific desmosomal proteins are varied. Plakoglobin promotes FN gene expression in keratinocytes, whereas Plakophilin-2 and Desmoplakin inhibit expression of FN and Collagen in cardiac myocytes (Todorović et al., 2010; Dubash et al., 2016). Mutations in Dsg2 have also been linked to fibrotic deposition in other contexts such as mouse models of arrhythmogenic cardiomyopathy (Krusche et al., 2010). Our work showing that Dsg2 controls both ECM production and MMP expression suggests that this desmosomal cadherin can act as an important coordinator of wound healing processes in keratinocytes.

A striking aspect of our findings is that while loss of Dsg2 triggers enhanced ECM and MMP expression, loss of Dsc2 is unable to do so. This specificity likely stems from the structural differences between these desmosomal cadherins. Dsg2 makes calcium-dependent homophilic interactions in the extracellular space, as well as heterophilic interactions with Dsc2 and classical cadherins such as E-cadherin and N-cadherin (Fuchs et al., 2022). Dsg2 and Dsc2 are expressed in all tissues which have desmosomes, namely simple epithelia, complex epithelia (basal layer) and the myocardium (Schäfer et al., 1996; Garrod et al., 2002; Dusek et al., 2007). Dsg and Dsc are 30% homologous, with the largest differences residing in their cytoplasmic tails. In contrast to Dsc, the intracellular tails of Dsg contain a desmoglein unique region (DUR) containing a proline-rich linker (PL), repeating unit domains (RUD1-6) and a terminal domain (TD). While studies have explored protein binding partners of the desmoglein-specific domains of the DUR, their cellular functions are still not well defined (Chen et al., 2002; 2012; Bonné et al., 2003). Exploring the structural and mechanistic differences between Dsg2 and Dsc2 signaling in controlling NF-κB signaling and ECM/MMP gene expression poses an interesting avenue of exploration for future studies. Additionally, the cell-type specificity we observed with robust effects in keratinocytes but not in MCF7 epithelial cells suggests that this Dsg2-NF-κB pathway may be adapted for the physiological demands of epithelia where mechanical stress, barrier function, and wound repair are critical responses.

Regulation of the production and secretion of ECM proteins is particularly important for the control of cell migration, whether during regulated processes of wound healing in the epidermis or during dysregulated migration in cancer (Urbanczyk et al., 2019; Selig et al., 2020; Walma and Yamada, 2020). While the mechanics of cell migration is fairly well understood, the myriad signaling nodes used by cells to communicate with their neighbors and the extracellular environment during this process is still an evolving field (Raja et al., 2007; Pascalis and Etienne-Manneville, 2017). Different desmosomal proteins have been ascribed both pro- and anti-migratory functions, suggesting that their effects are not simply a consequence of loss of cell-cell adhesion, but instead contribute complex cell type-specific signaling roles to the processes of cell migration (Raja et al., 2007; Bass-Zubek et al., 2009; Huber and Petersen, 2015). Several members of the desmosome complex (including Dsg2) have been shown to control the dynamics of the actin cytoskeleton and focal adhesions which are necessary for spreading and migration (Godsel et al., 2010; Todorović et al., 2010; Koetsier et al., 2014; Pascalis and Etienne-Manneville, 2017; Nekrasova et al., 2018).

The role of Dsg2 in cell migration is particularly complex and seemingly cell-context dependent, as prior studies have ascribed both pro-migratory and anti-migratory functions to this desmosomal cadherin (Min et al., 2023). Overexpression of Dsg2 has been shown to enhance proliferation and migration via activation of EGFR and cSrc (Overmiller et al., 2016). In contrast, several other studies have shown that loss of Dsg2 enhances invasion and migration via either EGFR, Erk, Src signaling or other mechanisms (Peitsch et al., 2014; Hütz et al., 2017; Lee et al., 2020). Dsg2 knockdown has also been shown to induce epithelial to mesenchymal transition in gall bladder cancer cells (Wang et al., 2022), and pro-EMT transcription factors (Snail) have been shown to increase Dsg2 degradation (Kume et al., 2013). Adding to the complex picture of the role of desmosomal cadherins in coordinating cell migration, Dsg3 has also been shown to have either pro- or anti-migratory effects. This inconsistency of findings indicates that individual desmosomal proteins have unique context-dependent signaling roles, providing a strong rationale for continued investigation into the mechanisms via which desmosomal proteins affect cell migration. The enhanced migration we observed in Dsg2-deficient cells, which was rescued by both NF-κB and MMP inhibition, further supports the functional relevance of this signaling pathway for keratinocyte behavior during wound healing.

The connection we show here between Dsg2 and inflammatory signaling can have important implications for understanding skin pathophysiology. Inflammatory skin conditions such as psoriasis are characterized by elevated NF-κB signaling, increased fibronectin expression, and barrier dysfunction. (Goldminz et al., 2013; Kobielak and Boddupally, 2014; Gubán et al., 2016). Prior work has shown that normal NF-κB signaling is important for immune homeostasis, as any perturbation of NF-κB signaling (whether overexpression or inhibition) in the skin results in acute inflammation (Pasparakis, 2009; 2012). Our findings suggest that dysregulation of Dsg2 expression or function could contribute to these pathological changes by inappropriately activating inflammatory and matrix remodeling pathways. Interestingly, inflammatory signaling can also lead to proteolytic cleavage of Dsg2 and compromised barrier integrity (Kamekura et al., 2015), suggesting a potentially pathological positive feedback loop between desmosome disruption and inflammation. Understanding how to interrupt this cycle could provide new therapeutic opportunities for inflammatory skin diseases.

The Dsg2-NF-κB signaling axis described here represents an important complement to our previous findings showing that Dsg2 controls cell-matrix interactions through Rap1-dependent focal adhesion signaling proteins (Shelton et al., 2021). Together, these studies reveal a sophisticated regulatory network where Dsg2 coordinates both inside-out signaling (affecting focal adhesion dynamics) and outside-in mechanisms (controlling ECM production and remodeling). This dual control mediated by Dsg2 allows keratinocytes to simultaneously modulate their adhesive interactions with the existing matrix while actively remodeling their extracellular environment. The integration of cell-cell and cell-matrix signaling through Dsg2 highlights the central role of desmosomes as signaling hubs that coordinate multiple aspects of cellular behavior beyond simple cell-cell adhesion.

In conclusion, our work has highlighted a previously uncharacterized signaling connection between Dsg2 and NF-κB which deepens our understanding of the crosstalk between cell-cell junctions and cell-matrix attachment in keratinocytes. Ultimately, our findings help redefine desmosomes from passive adhesive structures to dynamic inflammatory signaling platforms important for regulation of ECM expression, keratinocyte migration, processes of wound healing and inflammatory skin conditions.

## Data Availability

The datasets presented in this study can be found in online repositories. The names of the repository/repositories and accession number(s) can be found in the article/Supplementary Material.

## Abbreviations

Dsg2, Desmoglein-2; NF-κB, Nuclear factor-kappa B; MMP, Matrix Metalloproteinase; ECM, Extracellular Matrix; FN, Fibronectin.
