# Supplementary material for: The desmosomal cadherin Desmoglein-2 controls extracellular matrix expression and remodeling via NF-κB signaling in keratinocytes
Source: Front Cell Dev Biol. 2025 Oct 30;13:1691260. doi: 10.3389/fcell.2025.1691260 (PMC12611794; doi:10.3389/fcell.2025.1691260)

SUPPLEMENTARY INFORMATION

Supplementary Table S1:

The source of all primary and secondary antibodies used in this study are listed here, as well as dilutions used for western blots (WB) or immunofluorescence (IF). The protocols used for immunofluorescence and western blotting with these antibodies are detailed in the methods section.

Antibody	Source/Vendor	Catalog Number	Dilution
anti-Desmoplakin 1+2 (DP2.15)	Abcam	AB16434	WB 1:1000
Anti-Collagen III	Abcam	AB7778	IF 1:200
anti-p120 catenin	BD Biosciences	610134	WB 1:2000
anti-Plakoglobin	BD Biosciences	610253	WB 1:2000
anti-β catenin (D10A8)	Cell Signaling Technologies	8480	WB 1:2000
anti-E-cadherin (24E10)	Cell Signaling Technologies	3195	WB 1:2000
anti-NF-κB p65/RelA (D14E12)	Cell Signaling Technologies	8242	WB 1:1000<br>IF 1:200
anti-Fibronectin	Millipore Sigma	F3648	WB 1:2000<br>IF 1:400
anti-Plakophilin-2	Progen	651101	WB 1:500
anti-Tubulin	Santa Cruz Biotechnology	Sc-58884	WB 1:2000
anti-Desmoglein 2 (6D8)	ThermoFisher	326100	WB 1:1000
anti-Desmocollin 2/3 (7G6)	ThermoFisher	326200	WB 1:500
Anti-Desmoglein 3 (5G11)	ThermoFisher	326300	WB 1:500
Peroxidase-conjugated AffiniPure Goat anti-mouse IgG	Jackson ImmunoResearch	115-035-146	WB 1:10,000
Peroxidase-conjugated AffiniPure Goat anti-rabbit IgG	Jackson ImmunoResearch	111-035-144	WB 1:10,000
AlexaFluor 488-conjugated AffiniPure Donkey anti-rabbit IgG	Jackson ImmunoResearch	711-545-152	IF 1:400
Rhodamine Red X-conjugated AffiniPure Donkey anti-rabbit IgG	Jackson ImmunoResearch	711-295-152	IF 1:400

Supplementary Table S2:

For quantitative real-time PCR (qPCR), the following gene-specific forward and reverse primers were used (purchased from Integrated DNA Technologies). The protocol used for qPCR is included in the methods section.

Gene	Forward Primer	Reverse Primer
FN1	GTGGCAGAAGGAATATCTCGG	GAGAATACTGGTTGTAGGACTGG
COL1A1	CCCCTGGAAAGAATGGAGATG	TCCAAACCACTGAAACCTCTG
COL3A1	CTACTTCTCGCTCTGCTTCATC	CACAGACACATATTTGGCATGG
TGM2	TCAGCTACAATGGGATCTTGG	AAGGCAGTCACGGTATTTCTC
MMP1	TTTGGCTTCCCTAGAACTGTG	GCTATCATTTTGGGATAACCTGG
MMP3	AAGCTCTGAAAGTCTGGGAAG	CAGGTCCATCAAAAGGGTAAAAG
MX1	CAGCACCTGATGGCCTATCA	ACGTCTGGAGCATGAAGAACTG
MX2	AAACTGTTCAGAGCACGATTGAAG	ACCATCTGCTCCATTCTGAACTG
IFIT1	GCAGCCAAGTTTTACCGAAG	GCCCTATCTGGTGATGCAGT
IFIT3	AGTCTAGTCACTTGGGGAAAC	ATAAATCTGAGCATCTGAGAGTC
IL6	CCACTCACCTCTTCAGAACG	CATCTTTGGAAGGTTCAGGTTG
STAT1	TGAACTTACCCAGAATGCCC	CAGACTCTCCGCAACTATAGTG
GAPDH	ACATCGCTCAGACACCATG	TGTAGTTGAGGTCAATGAAGGG

Supplementary Figure Legends:

Supplementary Figure S1: *Knockdown of Dsg2 in other epithelial cell types (MCF7) does not induce elevation of ECM/MMP expression or NF-κB signaling.*

(A) Total RNA was isolated from MCF7 cells transfected with siCT or siDsg2, followed by qPCR to analyze mRNA levels of *FN1, COL3A1 and MMP1.* **(B)** siCT and siDsg2 MCF7 cells were processed for SDS-PAGE and blotted for the following proteins: Fibronectin, Dsg2 and Tubulin (loading control). **(C)** Total RNA was isolated from MCF7 cells transfected with siCT or siDsg2, followed by qPCR to analyze mRNA levels of *MX1* and *IFIT1*. All graphs shown represent fold change values of mRNA levels, with error bars indicating s.d. (N.S. = not significant).

Supplementary Figure S2: *Analysis of wound healing in A431CT and Dsg2KO cells.*

Control A431 cells (A431CT) and Dsg2 knockout cells (Dsg2KO) were grown to confluency within culture inserts (Ibidi). Lifting of the culture insert results in the creation of a uniform wound area in all samples (0 hr), followed by analysis of remaining wound area at 12 hr. Example images of 0 vs. 12 hr samples (which have been fixed and stained with AlexaFluor 568-tagged Phalloidin) are shown here to demonstrate the uniformity of the wound area at 0 hr.

Supplementary Figure S3: *TGF-β, Rap1 or RhoA/SRF signaling does not control ECM gene expression via Dsg2.*

Desmoglein-2 knockout (Dsg2KO) cells growing in culture were treated with either DMSO (vehicle control) or the following inhibitors: **(A)** SB431542 (5 μM) or GGTI-298 (10 μM); **(B)** Y27632 (1 μM) or CCG1423 (1 μM). 24 hours post-treatment, total RNA was isolated from DMSO or inhibitor-treated Dsg2KO cells, followed by qPCR to analyze mRNA levels of *FN1*. The graph shown represents fold change values of mRNA levels normalized to the DMSO vehicle control (blue reference line), with the error bars indicating s.d. (N.S. = not significant, * = $p < 0.05$).

Supplementary Figure S4: *Loss of Dsg2 in A431 cells results in enhanced expression of NF-κB target genes.*

Total RNA was isolated from A431CT and Dsg2KO cells, followed by qPCR to analyze mRNA levels of *MX1, MX2, IFIT1, IFIT3, IL6,* and *STAT1*. Graphs shown represent fold change values of mRNA levels, with error bars indicating s.d. (*** = $p < 0.001$).

Supplementary Figures S5-6: *Unprocessed blot images.*

Unprocessed images for western blot data in all figures are shown here. To blot for several proteins of different molecular weights, membranes were frequently cut into different segments using the molecular weight ladder as a guide, and individual segments probed with different antibodies. For example, this approach allowed us to confirm equal loading of all gels by probing for a reference protein (Tubulin) on all membranes.

Supplementary Figure S1, Hunter SE *et. al.*

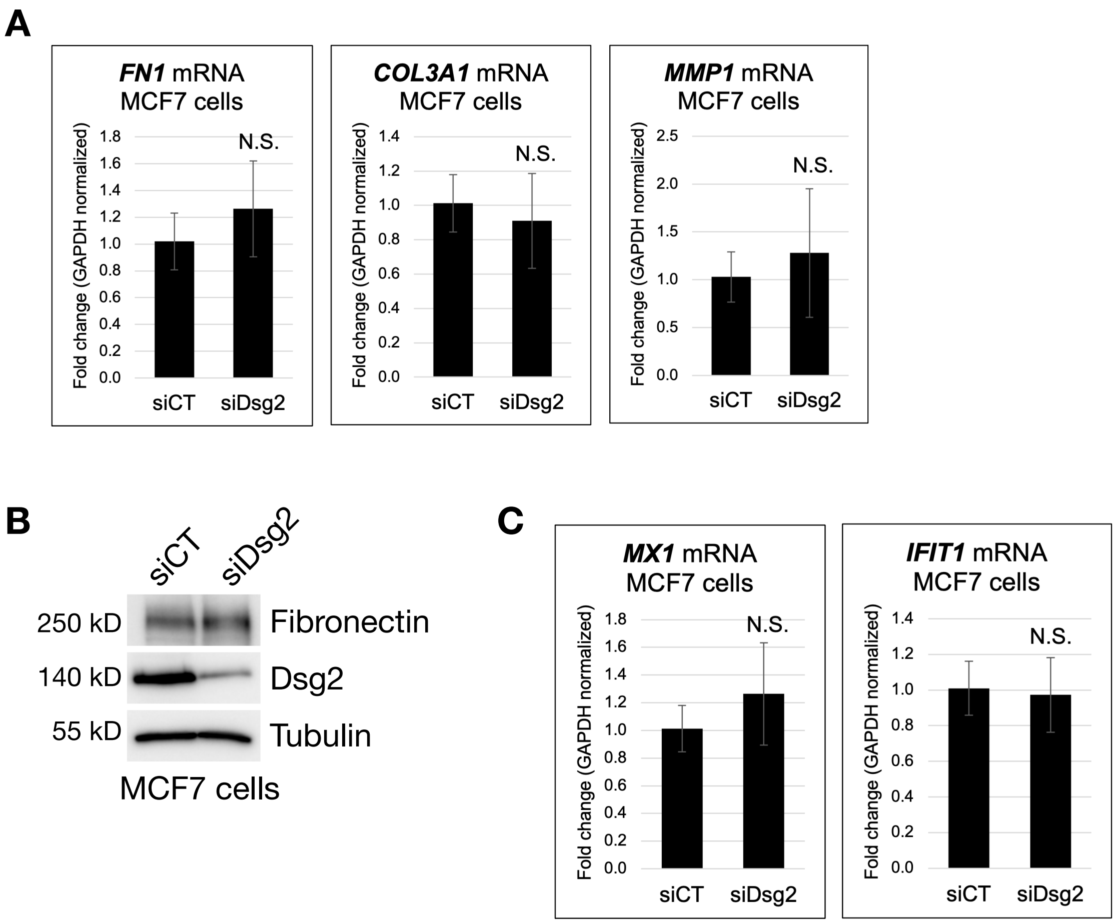

Supplementary Figure S2, Hunter SE *et. al.*

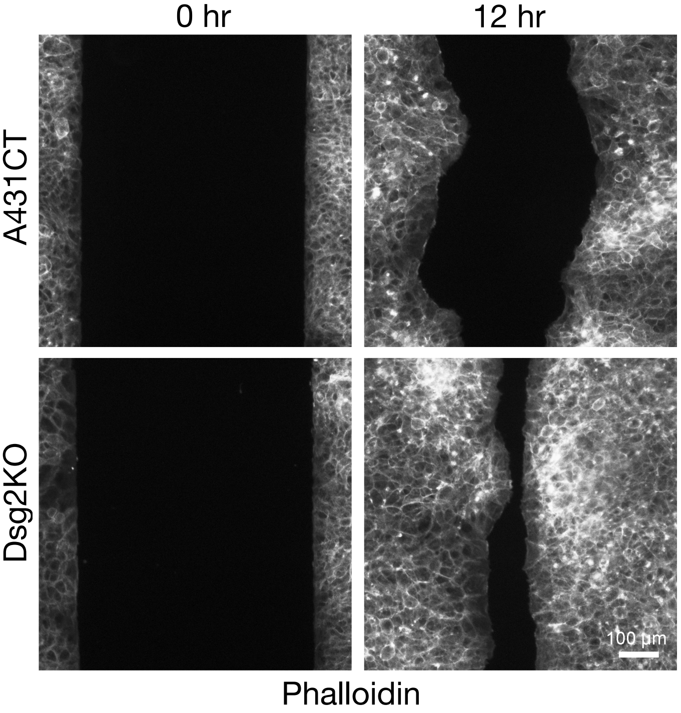

Supplementary Figure S3, Hunter SE *et. al.*

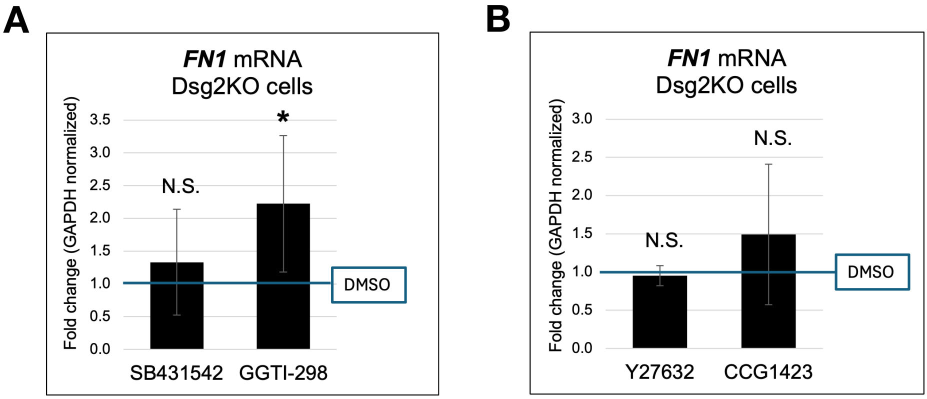

Supplementary Figure S4, Hunter SE *et. al.*

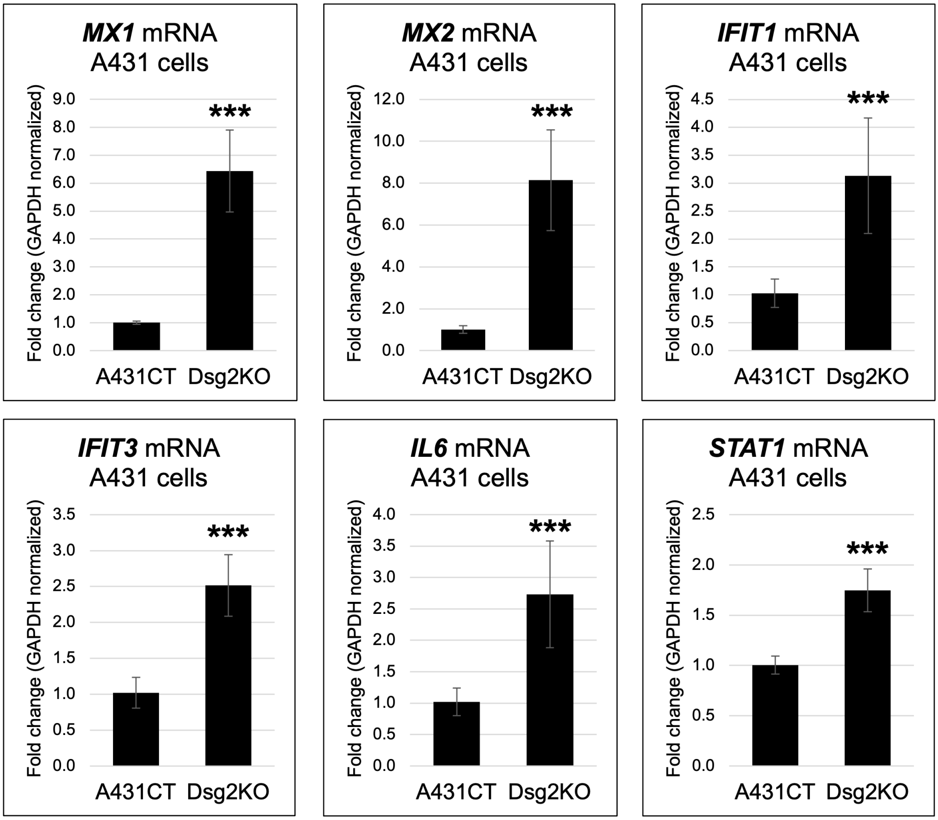

Supplementary Figure S5, Hunter SE *et. al.*
Unprocessed blot images

Figure 1B: HaCaT cells

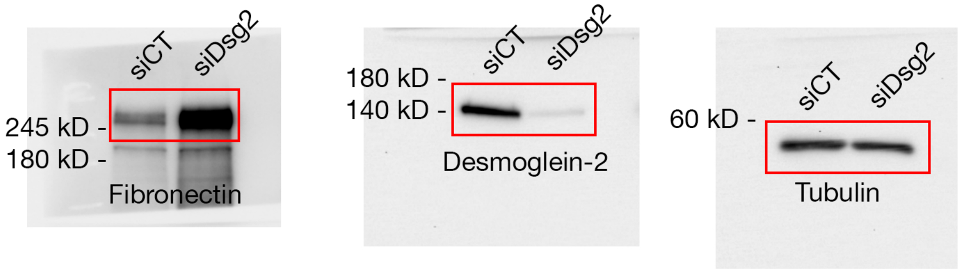

Figure 1D: A431 cells

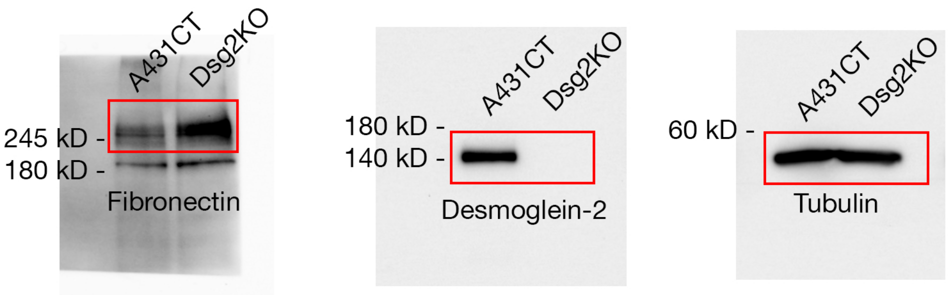

Figure 1E: HaCaT cells

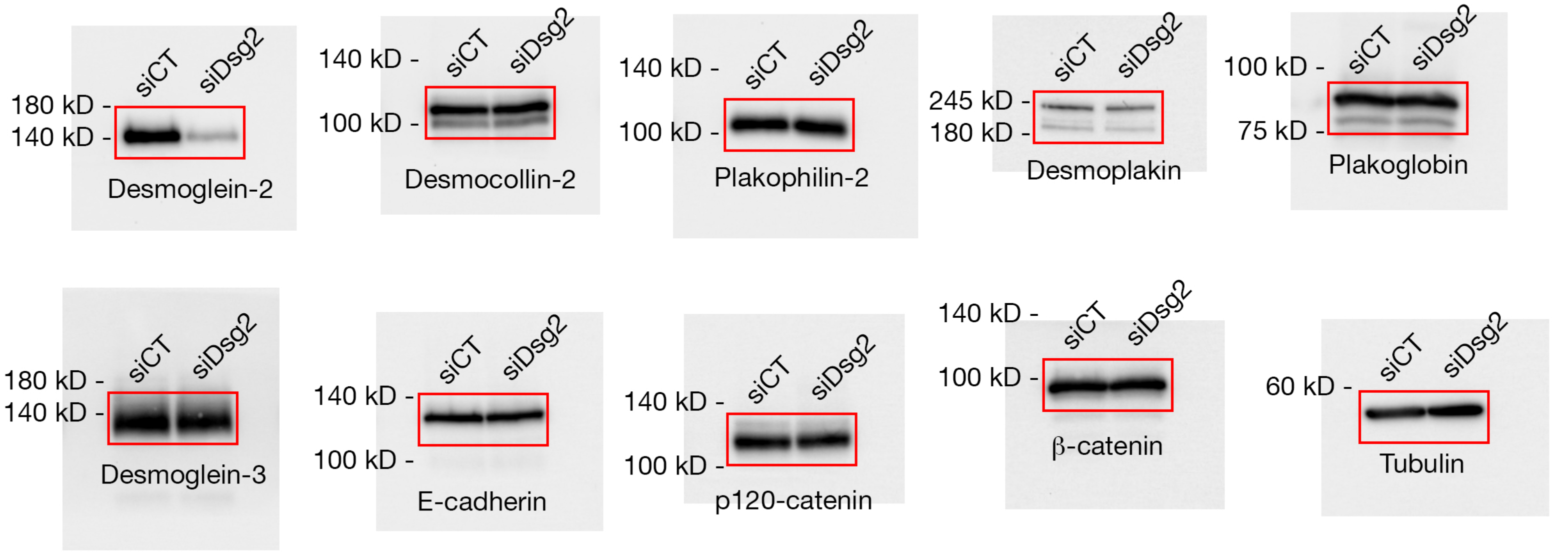

Figure 1F: HaCaT cells

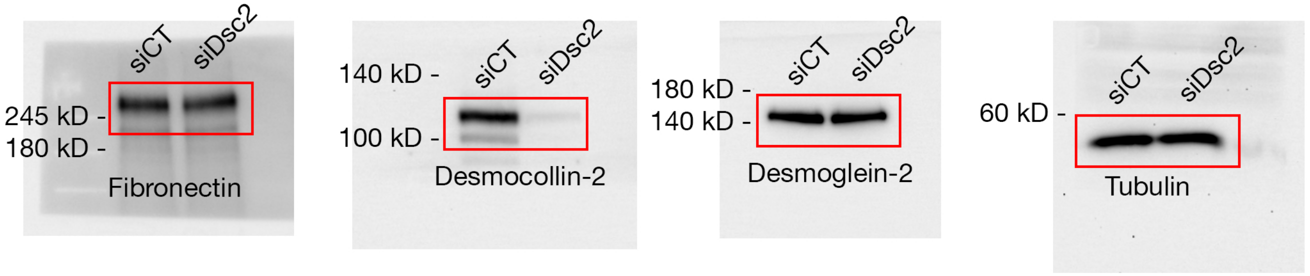

Supplementary Figure S6, Hunter SE *et. al.*

Unprocessed blot images

Figure 2B: SCC9 cells

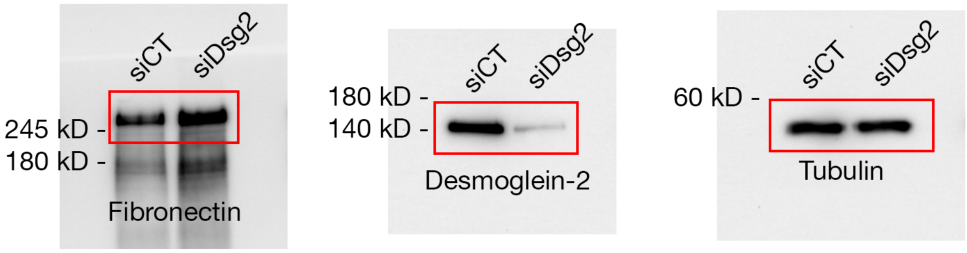

Figure 6D: HaCaT cells

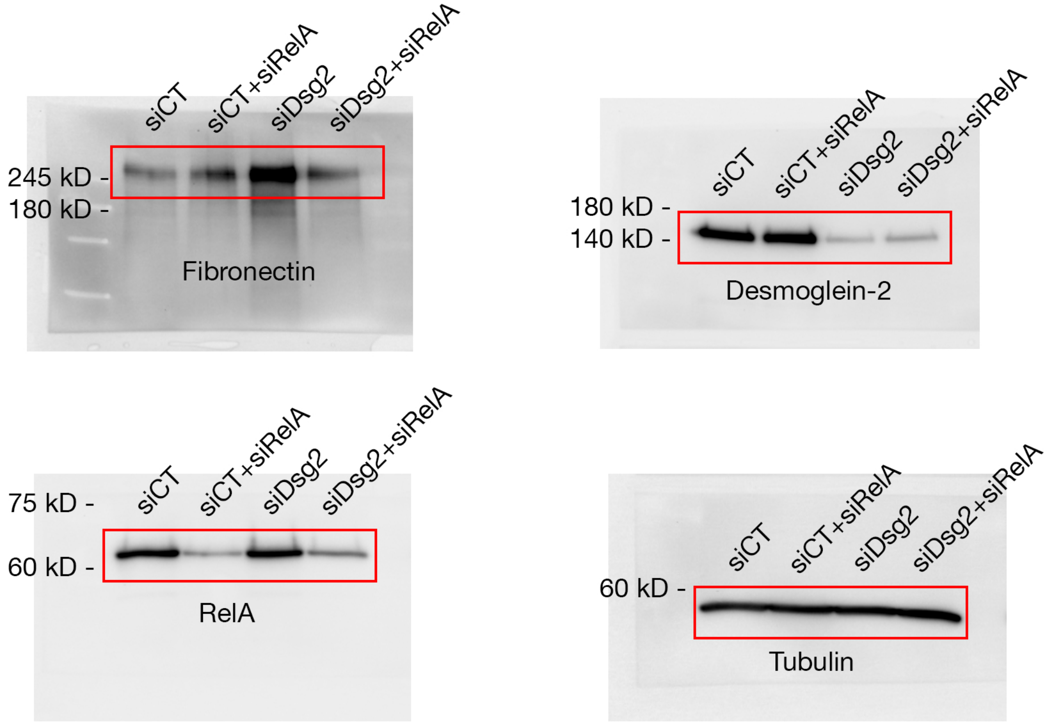

Supplementary Figure S1B: MCF7 cells

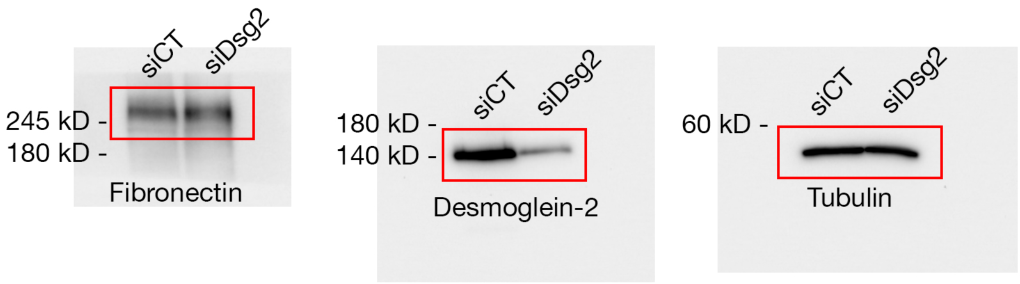

Supplement: Supplementary file 1 [file DataSheet1.pdf]
